# The Effects of Virtual Reality in Maternal Delivery: Systematic Review and Meta-analysis

**DOI:** 10.2196/36695

**Published:** 2022-11-23

**Authors:** Nuo Xu, Sijing Chen, Yan Liu, Yuewen Jing, Ping Gu

**Affiliations:** 1 School of Nursing Nanjing Medical University Nanjing China

**Keywords:** virtual reality technology, delivery, labor pain, anxiety, meta-analysis, systematic review, pain, pregnancy, virtual reality, maternity, labor, technology, pregnant women, review, childbirth, mental health

## Abstract

**Background:**

Extreme labor pain has negative effects; pharmacologic analgesic modalities are effective but are accompanied by adverse effects. Virtual reality (VR) works as a distracting nonpharmacologic intervention for pain and anxiety relief; however, the effects of VR use in laboring women is unknown.

**Objective:**

Our study aimed to determine the safety and effectiveness of VR technology during labor and delivery and investigate whether it impacts labor and patient satisfaction.

**Methods:**

In all, 7 databases (PubMed, Embase, Web of Science, the Cochrane Library, CINAHL, China National Knowledge Infrastructure, and Wan-Fang Database) were systematically searched for randomized controlled trials of VR use in pregnancy and childbirth from the time of database construction until November 24, 2021. Two researchers extracted data and evaluated study quality using the Cochrane Risk of Bias tool 2.0. Outcome measures were labor pain, anxiety, duration, satisfaction, and adverse events. Meta-analyses were performed where possible.

**Results:**

A total of 12 studies with 1095 participants were included, of which 1 and 11 studies were rated as “Low risk” and “Some concerns” for risk of bias, respectively. Of the 12 studies, 11 reported labor pain, 7 reported labor anxiety, and 4 reported labor duration. Meta-analysis revealed that VR use could relieve pain during labor (mean difference –1.81, 95% CI –2.04 to –1.57; *P*<.001) and the active period (standardized mean difference [SMD] –0.41, 95% CI –0.68 to –0.14; *P*=.003); reduce anxiety (SMD –1.39, 95% CI –1.99 to –0.78; *P*<.001); and improve satisfaction with delivery (relative risk 1.32, 95% CI 1.10-1.59; *P*=.003). The effects of VR on the duration of the first (SMD –1.12, 95% CI –2.38 to 0.13; *P*=.08) and second (SMD –0.22, 95% CI –0.67 to 0.24; *P*=.35) stages of labor were not statistically significant.

**Conclusions:**

VR is safe and effective in relieving maternal labor pain and anxiety; however, due to the heterogeneity among studies conducted to date, more rigorous, large-scale, and standardized randomized controlled trials are required to provide a higher-quality evidence base for the use of VR technology in maternal labor, with the aim of improving experience and outcomes.

**Trial Registration:**

PROSPERO CRD42021295410; https://www.crd.york.ac.uk/prospero/display_record.php?RecordID=295410

## Introduction

The pain of labor is the highest pain level, lasts longer than acute pain, and occurs during all 3 stages of labor (dilation of the uterus, delivery of the fetus, and delivery of the placenta) [1]. Although labor pain occurs naturally, extreme pain can lead to negative physiological changes during labor such as excessive neuroendocrine stress, maternal acidemia, and prolonged labor [2,3]. Therefore, a reasonable reduction in pain intensity and duration, within safe limits, is necessary. Although epidural analgesia (the most commonly used form of pain relief in labor) has been shown to be safe and effective in this context, it is associated with longer labor times and more surgical interventions [4]. In addition, opioids such as pethidine reduce labor pain but increase maternal drowsiness, nausea, and vomiting [5] and can even cause respiratory depression [6]. Moreover, pharmacological analgesia fails to address cognitive and emotional factors, which significantly influences pain and anxiety. Thus, the World Health Organization recommends the use of nonpharmaceutical methods of pain relief [7].

Some nonpharmacological methods of treating pain, such as music [8] and aromatherapy [9], have been developed to reduce the use of analgesic drugs, but suffer from the disadvantages of inconvenience and a precipitous learning curve [10].

Distraction is a common intervention during medical procedures and is effective in reducing pain and anxiety [11]. As an integrated distraction technology, combined with computer technology, virtual reality (VR)—creating an immersive, interactive, and imaginative 3D virtual environment—has the potential to distract people from external stimuli and enhance positive thinking [12]. VR allows user to interact with a realistic 3D virtual environment by stimulating multiple perceptions, altering the activity of the complex physiological pain modulation systems by dividing attentional tasks to reduce the level of attention to pain [12-14]. Increasing evidence supports VR as an effective distraction intervention that is a safe and effective alternative strategy for treating adults [15] and children [16], burns [17], and acute pain [18]; however, labor pain differs from other types of pain, in that it is associated with strong emotions and varies in intensity as labor progresses. Pain during uterine contractions is intermittent, whereas persistent pain is associated with generalized injuries [3]. Hence, although VR is also an effective treatment for chronic pain [15], it is not appropriate to extrapolate the findings of meta-analyses addressing the ability of VR to relieve general pain to maternal labor.

Due to the limitations of VR equipment and the number of experiments, clinical trials to date have been small-scale, and differences in experimental design have contributed to controversial findings. Thus, it is essential to evaluate the effectiveness of VR in maternal delivery, but, to our knowledge, there has been no previous systematic review that specifically focused on this issue. Further, Chinese scholars have made specific contributions to this field in recent years, and their work deserves attention.

The purpose of this review was to investigate the effectiveness and safety of using VR as a method of relieving maternal anxiety and pain. Our results will contribute to clinical practice and justify the investment in equipment used in maternity hospitals.

## Methods

### Overview and Registration

This systematic review conformed to the PRISMA (Preferred Reporting Items for Systematic Reviews and Meta-Analyses) statement [19] and was registered in advance in the international Prospective Register of Systematic Review database (registration number CRD42021295410).

### Inclusion and Exclusion Criteria

The Population, Intervention, Comparison, Outcomes, and Study design model was used to establish the article inclusion criteria, as follows:

- Population: women aged 18-35 years, who were at >34 weeks of gestation, with a normal fetus and no pregnancy complications, able to cooperate with the study and give informed consent
- Intervention: any type of VR-based interventions, including unrestricted VR equipment and contents
- Comparison: traditional methods (such as closed observation of maternal vital signs and fetal heart rate, explanation of labor- and delivery-related precautions, nutritional guidance, and psychological care) or nonintervention
- Outcomes: primary outcomes include labor pain and anxiety; secondary outcomes include labor progress, labor satisfaction, and adverse events; no restrictions on the assessment tools
- Study design: randomized controlled trials (RCTs), case-controlled trials, and quasi-experimental studies

Studies were excluded if they were (1) reviews, animal experiments, unfinished experiments, conference papers, or study protocols; (2) not in English or Chinese; or (3) evaluated as “High risk” for risk of bias.

### Search Strategy

PubMed, Embase, Web of Science, the Cochrane Library, CINAHL, China National Knowledge Infrastructure, and Wan-Fang databases were comprehensively searched for relevant literature. Studies must have been published before November 24, 2021.

Search terms were classified into 2 groups: (1) “Virtual Reality,” “Virtual Reality Exposure Therapy,” “User Computer Interface,” and “Augmented Reality”; and (2) “Pregnant Woman,” “Deliveries,” “Obstetric,” and “Parturition.” Words in each group were linked by “OR” and searched with the other group by “AND.” The databases were also searched using a combination of free words and subject word forms. Additional studies within 20 years were identified from the reference lists of the screened articles. Details of the PubMed database search strings are displayed in Textbox 1. Full details of the final search strategies for each database are available in Multimedia Appendix 1.

Two researchers (NX and SC) screened the studies independently. First, articles were imported into Endnote X9 software (Clarivate) to remove duplicates. Titles and abstracts were then examined, followed by a careful reading of the full text and further selection according to the inclusion and exclusion criteria. Finally, the result of screenings conducted by the 2 individuals were cross-checked.

**PubMed search strategies.**

((((((((((((“Pregnant Women”[Mesh]) OR (Woman, Pregnant[Title/Abstract])) OR (Women, Pregnant[Title/Abstract])) OR (“Delivery, Obstetric”[Mesh])) OR (Obstetric Deliveries[Title/Abstract])) OR (Obstetric Delivery[Title/Abstract])) OR (“Parturition”[Mesh])) OR (Parturitions[Title/Abstract])) OR (Birth[Title/Abstract])) OR (Births[Title/Abstract])) OR (Childbirth[Title/Abstract])) OR (Childbirths[Title/Abstract])) AND (((((((((((((((((((((((((((((((((((((((((“Virtual Reality”[Mesh] OR “Virtual Reality Exposure Therapy”[Mesh]) OR (Virtual Reality, Educational[Title/Abstract])) OR (Educational Virtual Realities[Title/Abstract])) OR (Educational Virtual Reality[Title/Abstract])) OR (Reality, Educational Virtual[Title/Abstract])) OR (Virtual Realities, Educational[Title/Abstract])) OR (Virtual Reality, Instructional[Title/Abstract])) OR (Instructional Virtual Realities[Title/Abstract])) OR (Instructional Virtual Reality[Title/Abstract])) OR (Realities, Instructional Virtual[Title/Abstract])) OR (Reality, Instructional Virtual[Title/Abstract])) OR (Virtual Realities, Instructional[Title/Abstract])) OR (Virtual Reality Immersion Therapy[Title/Abstract])) OR (Virtual Reality Therapy[Title/Abstract])) OR (Reality Therapies, Virtual[Title/Abstract])) OR (Reality Therapy, Virtual[Title/Abstract])) OR (Therapies, Virtual Reality[Title/Abstract])) OR (Therapy, Virtual Reality[Title/Abstract])) OR (Virtual Reality Therapies[Title/Abstract])) OR (“Augmented Reality”[Mesh])) OR (Augmented Realities[Title/Abstract])) OR (Realities, Augmented[Title/Abstract])) OR (Reality, Augmented[Title/Abstract])) OR (Mixed Reality[Title/Abstract])) OR (Mixed Realities[Title/Abstract])) OR (Realities, Mixed[Title/Abstract])) OR (Reality, Mixed[Title/Abstract])) OR (“User－Computer Interface”[Mesh])) OR (Virtual System[Title/Abstract])) OR (Interface, User－Computer[Title/Abstract])) OR (Interfaces, User－Computer[Title/Abstract])) OR (User－Computer Interfaces[Title/Abstract])) OR (Interfaces, User Computer[Title/Abstract])) OR (User Computer Interfaces[Title/Abstract])) OR (Interface, User Computer[Title/Abstract])) OR (Virtual Systems[Title/Abstract])) OR (System, Virtual[Title/Abstract])) OR (Systems, Virtual[Title/Abstract])) OR (virtual environment[Title/Abstract])) OR (immersion VR[Title/Abstract])) OR (Reality, Virtual[Title/Abstract]))

### Data Extraction

The basic characteristics of the included studies, including author, country, year, sample size, age, intervention, and outcome indicators were independently extracted into Microsoft Excel 2016 by 1 reviewer (NX) and checked for correctness by another (SC). Attempts were also made to contact the corresponding authors for more information about studies for which results were not reported. Any discrepancies were discussed in a consensus meeting with all the reviewers.

### Quality Assessment

Evaluation was conducted independently by the 2 researchers (NX and SC), and disagreements were resolved by consensus. Study quality and risks of bias were assessed using the Cochrane Collaboration’s tool (Risk of Bias tool 2.0) for assessing risk of bias in randomized trials [20]. The Cochrane Risk of Bias tool 2.0 assesses 5 domains: bias in the randomization process, bias in deviation from established interventions, bias in outcome measurement, bias of missing ending data, and bias in selective reporting of results. Items were categorized as “Low risk,” “High risk,” or “Some concerns.” Overall risk was assessed as “Low risk” if all 5 domains were assessed as low risk and as “High risk” if any domain was assessed as high risk; all other RCTs were assessed as “Some concerns.”

### Data Synthesis and Analysis

Data were analyzed using Review Manager (version 5.4; Cochrane Collaboration), and meta-analysis was performed if more than 2 studies had the same outcome and available data. The test level was set at α=.05.

For continuous outcomes, the mean difference (MD) with 95% CI was calculated when outcome measurements in all studies were made on the same scale. Standardized mean difference (SMD) was used when the studies did not yield directly comparable data [21]. Dichotomous variables were expressed as relative risk with 95% CI. The *I*^2^ statistic was used to determine whether there was heterogeneity among studies. If the heterogeneity was acceptable (chi-square *P*>.10, *I*^2^<50%), effect sizes were combined using a fixed-effects model; if the heterogeneity was large (chi-square *P*≤.10, *I*^2^≥50%), subgroup analysis was performed; and when no significant clinical heterogeneity existed, a random-effects model was used [22]. A qualitative review was also performed when studies could not be included in the meta-analysis. In cases where outcome indicators were not combined for meta-analysis or only 1 study reported an outcome, a narrative approach was applied for systematic review.

## Results

### Selection and Characteristics of Included Studies

**Figure 1:**
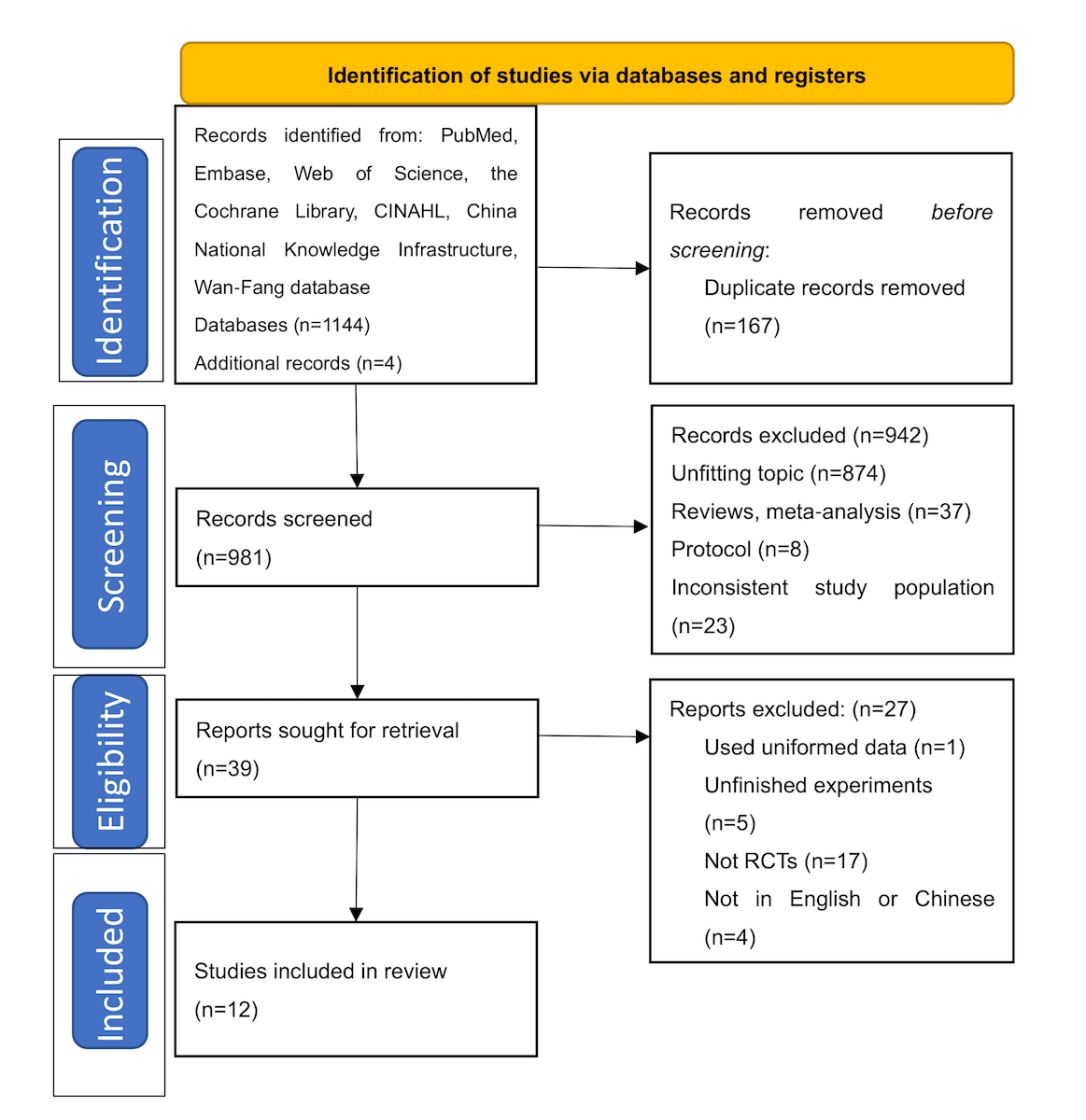
PRISMA (Preferred Reporting Items for Systematic Reviews and Meta-Analyses ) flowchart. RCT: randomized controlled trial.

According to the search strategy, 1144 studies were initially retrieved, and 4 additional articles were obtained by tracking references. After the removal of duplicates, the titles and abstracts of 981 studies were screened, and 942 studies were excluded because they were unrelated or not RCTs. The full text was checked for 39 studies, of which 1 was excluded for repeated publication, 5 were uncompleted experiments, and 21 studies did not meet the inclusion criteria. Finally, 12 studies were included in our systematic review (Figure 1) [23-34].

All included studies were published between 2019 and 2021, including 4 in Chinese and 8 in English. The studies were from China [24,25,32-34], the United States [26,30], Turkey [23,29], Iran [28,31], and the Netherlands [27]. Of the 12 included studies, 11 were RCTs [23-29,31-34] and 1 was a cross-over RCT [30]. In all, 6 of the studies used VR glasses [23,24,26,29,31,33], and 3 used head-mounted VR devices [28,30,32]. The basic characteristics of the included literature are presented in Table 1.

**Table 1:** Characteristics of studies included in this review.

Study, year	Country	Design	Age (years), mean (SD)		Sample size, n		Content	Intervention			Outcome^a^	Instrument
			VR^b^ group	Control group	VR group	Control group		Time	VR group	Control group		
Li et al [24], 2020	China	RCT^c^	24.53 (4.41)	25.6 (3.95)	35	36	Natural scenery	First stage of labor to end	R^d^ + E^e^ + G^f^	R	1, 2, and 4	VAS^g^, VRS^h^, PPI^i^, SAS^j^, and SDS^k^
Liang et al [34], 2020	China	RCT	27.69 (1.03)	27.47 (1.20)	30	30	Visual experience + music	First stage of labor to end	VR + R	R	1, 3, 5, and 6	VAS and EPDS^l^
Lin et al [33], 2021	China	RCT	26.24 (3.01)	27.03 (2.98)	66	48	Natural Scenery + music	Self-selected wear time, 5-60 min each time, full wear	G + music + R	R	1, 2, 3, and 4	VAS and SAS
Liu and Wan [32], 2020	China	RCT	28.72 (3.83)	27.37 (4.19)	80	80	Self-selection	VR experience 2 days after admission, 1 time per day for 2 h, worn again at the time of delivery	H^m^ + R	R	1 and 2	VAS, PPI, SAS, and PRI^n^
Wu et al [25], 2020	China	RCT	28.4 (4.58)		50	49	Self-selection	30 min after epidural paroxysmal	VR + R	R	1, 2, 4, and 5	STAI^o^ and NRS^p^
Akin et al [23], 2021	Turkey	RCT	27.23 (3.10)		50	50	Fetal image at 28 weeks	Intervention at delivery, mean 14.18 (SD 14.86) min	G + R	R	1, 2, and 3	VAS and PASS^q^
Ebrahimian and Rahmani Bilandi [31], 2021	Iran	RCT	24.23 (4.44)		31	31	Natural scenery + music	Use during the first and second stages of labor，20 min each time	G + R	R	3 and 4	MCSRS^r^
Frey et al [30], 2019	United States	Cross-RCT	27.9 (5.6)		27		Natural scenery + music	No more than 10 min	H + R	R	1 and 5	NRS
Gür and Apay [29], 2020	Turkey	RCT	1: 25.61 (5.14); 2: 25.89 (4.29); 3: 25.36 (4.54); 4: 27.65 (6.36)	5: 26.39 (4.32)	1: 54; 2: 55; 3: 55; 4: 55; 5: 54		1: newborn video photos + classical music; 2: video album; 3: a film introducing Turkey; 4: classical music	10 min	G + R	R	1	VAS, VRS, PPI, SAS, and SDS
Momenyan et al [28], 2021	Iran	RCT	28.41 (4.50)	30.37 (6.09)	26	26	Natural scenery + music	Performed 2 times, nearly 10 min each time	H + E + R	R	1, 2, 5, and 7	NRS and Apgar
Noben et al [27], 2019	The Netherlands	RCT	32.6 (3.9)	33.12 (4.3)	49	48	Informative video on cesarean delivery	Prenatal, unlimited time	Standard Information by VR videos	Standard Information	1, 2, and 5	VAS and TPDS^s^
Wong et al [26], 2021	United States	RCT	31.6 (5.6)	32.5 (3.6)	21	19	Natural scenery + music	30 min	G + R	R	1 and 5	PROMIS^t^ global health survey

^a^Outcomes: 1=pain, 2=anxiety, 3=time of delivery, 4=satisfaction, 5=adverse effects, 6=depression, and 7=newborn endings.

^b^VR: virtual reality.

^c^RCT: randomized controlled trial.

^d^R: routine obstetric care.

^e^E: earphones.

^f^G: VR glasses.

^g^VAS: Visual Analogue Scale.

^h^VRS: Verbal Rating Scale.

^i^PPI: Present Pain Index.

^j^SAS: Self-Rating Anxiety Scale.

^k^SDS: Self-Rating Depression Scale.

^l^EPDS: Edinburgh Postnatal Depression Scale.

^m^H: Head-mounted VR device.

^n^PRI: Pain Rating Index.

^o^STAI: Spielberger Trait Anxiety Inventory.

^p^NRS: Numerical Rating Scale.

^q^PASS: Perinatal Anxiety Screening Scale.

^r^MCSRS: Mackey Childbirth Satisfaction Rating Scale.

^s^TPDS: Tilburg Pregnancy Distress Scale.

^t^PROMIS: Patient Reported Outcomes Measurement Information System.

### Methodological Quality

All 12 articles had detailed inclusion and exclusion criteria and showed an acceptable risk of bias. For 6 studies [24,25,31-34], there was a possibility of bias in the randomization process, mostly because the distribution was unclear; only 1 study did not generate a random sequence [29]. Further, 11 studies were biased in deviation from established interventions [23-28,30-34], and all had low risk of bias in outcome measurement. Only 1 study did not lack outcome data [29], and 5 had unclear risk in selective reporting of results [24,25,32-34]. The risk of bias is summarized in Table 2.

**Table 2:** Risks of bias for the randomized controlled trials included in this study.

Author	Year	Risk of bias assessment					Overall bias
		Randomization process	Deviations from intended interventions	Measurement of the outcome	Missing outcome data	Selection of the reported result	
Li et al [24]	2020	Some concerns	High risk	Low risk	Some concerns	Some concerns	Some concerns
Liang et al [34]	2020	Some concerns	High risk	Low risk	Some concerns	Some concerns	Some concerns
Lin et al [33]	2021	Some concerns	High risk	Low risk	Some concerns	Some concerns	Some concerns
Liu and Wan [32]	2020	Some concerns	High risk	Low risk	Some concerns	Some concerns	Some concerns
Wu et al [25]	2020	Some concerns	High risk	Low risk	Some concerns	Some concerns	Some concerns
Akin et al [23]	2021	Low risk	High risk	Low risk	Some concerns	Low risk	Some concerns
Ebrahimian and Rahmani Bilandi [31]	2021	Some concerns	High risk	Low risk	Some concerns	Low risk	Some concerns
Gür and Apay [29]	2020	Low risk	Low risk	Low risk	Low risk	Low risk	Low risk
Momenyan et al [28]	2021	Low risk	High risk	Low risk	Some concerns	Low risk	Some concerns
Frey et al [30]	2019	Low risk	High risk	Low risk	Some concerns	Low risk	Some concerns
Noben et al [27]	2019	Low risk	High risk	Low risk	Some concerns	Low risk	Some concerns
Wong et al [26]	2021	Low risk	High risk	Low risk	Some concerns	Low risk	Some concerns

### Effects of VR

#### Effect of VR on Pain

In all, 4 studies explored the effect of VR on pain during childbirth [24,32-34], and another 3 explored the effect on pain reduction during the active period (when the uterus is 3-10 cm dilated) [23,26,29].

#### Effect of VR on Pain During Childbirth

**Figure 2:**
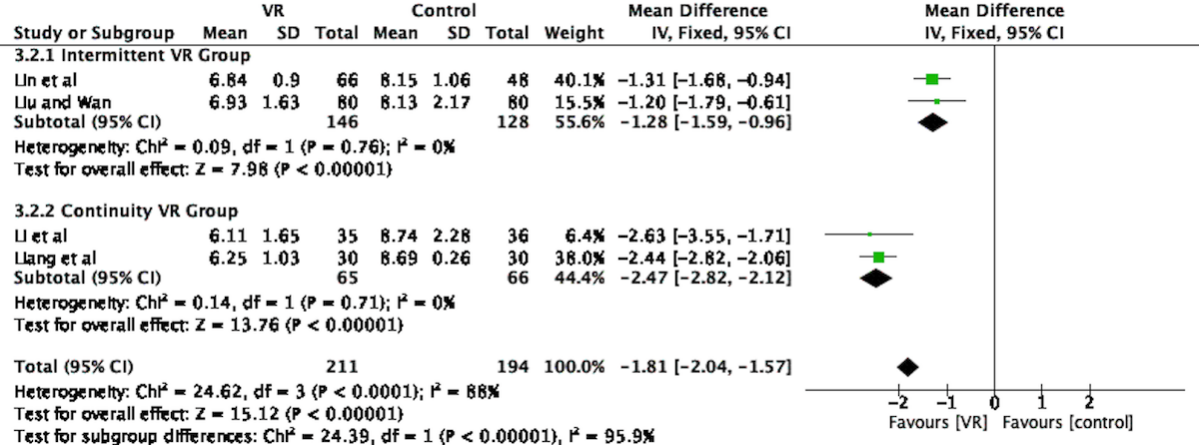
Forest plot of the effect of VR on pain during childbirth. IV: inverse variance; VR: virtual reality.

In all, 4 studies comprising 405 patients assessed the effect of VR on maternal pain during childbirth [24,32-34]. There was significant heterogeneity among the studies (chi-square *P<*.001; *I*^2^=88%) on analysis using MD. Therefore, we divided the 4 studies into a *continuity VR group* (where VR was used from the first stage until the end of labor) [24,34] and an *intermittent VR group* (where there were interruptions in the VR) [32,33] for subgroup analysis. There was extremely high heterogeneity between the 2 groups (chi-square *P*<.001; *I*^2^=95.9%) but no heterogeneity within the intermittent (chi-square *P*=.76; *I*^2^=0%) and continuity (chi-square *P*=.71; *I*^2^=0%) VR groups. Therefore, we use a fixed-effects model for analysis. As shown in Figure 2, all differences were significant (*P<*.001).

#### Effect of VR on Pain During the Active Period

**Figure 3:**
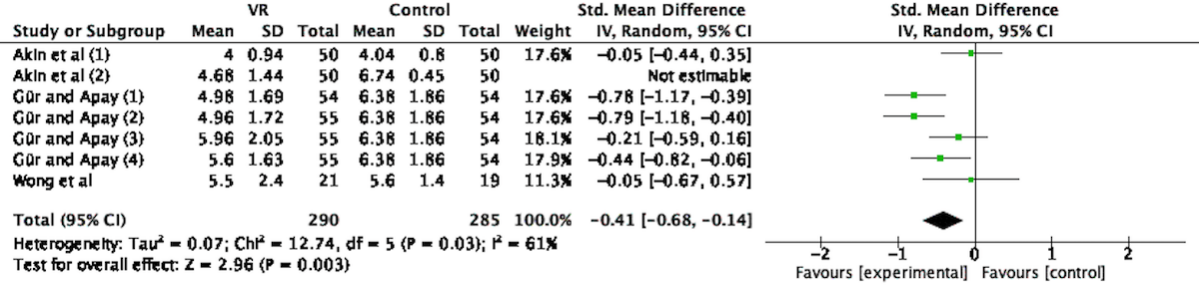
Forest plot for the effect of VR on pain during the active period. IV: inverse variance; VR: virtual reality.

In all, 3 studies (n=575 participants) applied VR during active labor [23,26,29]. Gür and Apay [29] divided subjects into 4 VR content groups: (1) newborn video photos and classical music, (2) video album, (3) a film introducing Turkey, and (4) classical music; each group was analyzed separately. Akin et al [23] explored the analgesic effects of VR at 4 cm (“Akin et al (1)”) and 9 cm (“Akin et al (2)”), which were included as 2 experiments. SMD and random-effects models were used because of the variation in pain assessment tools (Visual Analogue Scale and Numerical Rating Scale). We found high heterogeneity among studies, likely due to differences in the duration, schedule, intensity, and type of interventions and methodological factors, and performed a sensitivity analysis. After excluding Akin et al (2), the *I*^2^ value decreased from 87% to 61%; therefore, meta-analysis was performed on the remaining experiments and showed that VR relieved labor pain during the active period (SMD –0.41, 95% CI –0.68 to –0.14; *P*=.003; Figure 3).

#### Effect of VR on Labor Anxiety

**Figure 4:**
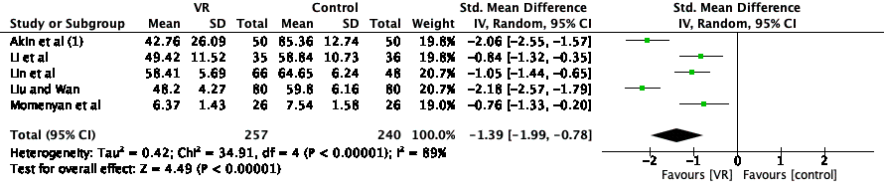
Forest plot for the effect of VR on labor anxiety. IV: inverse variance; VR: virtual reality.

In all, 7 studies described the effect of VR on maternal anxiety [23-25,27,28,32,33], and a meta-analysis was applied to 5 of them (n=497 participants) [23,24,28,32,33]. SMD was calculated and showed high heterogeneity among studies (chi-square *P*<.001; *I*^2^=89%). Sensitivity analysis demonstrated that the results were stable after excluding each single study; therefore, we speculated that the high heterogeneity was likely due to differences in the duration, schedule, intensity, and type of interventions and methodological factors and applied a random-effects model. Meta-analysis showed that VR reduced maternal anxiety during delivery (SMD –1.39, 95% CI –1.99 to –0.78; *P*<.001), as shown in Figure 4.

#### Effect of VR on the Process of Labor

**Figure 5:**
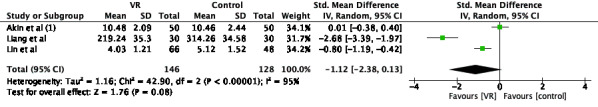
Forest plot for the effect of VR on duration of the first stage of labor. IV: inverse variance; VR: virtual reality.

**Figure 6:**
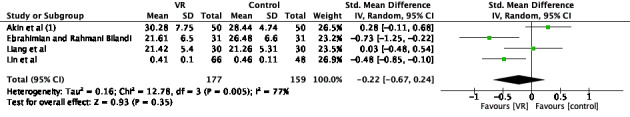
Forest plot for the effect of VR on duration of the second stage of labor. IV: inverse variance; VR: virtual reality.

In all, 3 studies (n=274) explored the duration of the first stage of labor [23,33,34], and 4 studies (n=336) included the second stage [23,31,33,34]. The calculation of SMD values, due to differences in measurements, and sensitivity analysis, because of the higher heterogeneity, generated stable results. The high heterogeneity may have been due to differences in measurement timing, methodology, and intervention protocols. The data presented in Figures 5 and 6 demonstrate that the effects of VR in reducing the duration of the first (SMD –1.12, 95% CI –2.38 to 0.13; *P*=.08) and second (SMD –0.22, 95% CI –0.67 to 0.24; *P*=.35) stages were not statistically significant.

#### Effect of VR on Labor Satisfaction

**Figure 7:**
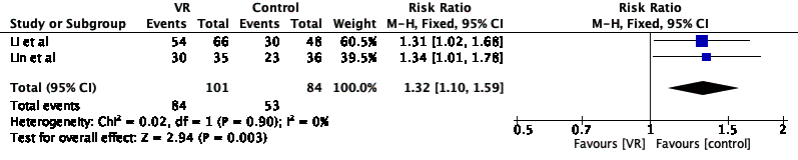
Forest plot of the effect of VR on labor satisfaction. M-H: Mantel-Haenszel; VR: virtual reality.

In all, 4 studies reported the effect of VR use on satisfaction with childbirth [24,25,31,33], and 2 (n=137) were subjected to meta-analysis [24,33], which showed significantly higher satisfaction with childbirth in the VR group (relative risk 1.32; 95% CI 1.10-1.59; *P*=.003; Figure 7). Similar results were reported by Wu et al [25] (*P*<.001) and Ebrahimian and Rahmani Bilandi [31] (*P*<.001).

#### Adverse Effects

In all, 6 studies reported adverse events [25-28,30,34], including nausea [25,30], vomiting [26], eye focus disorders [26,27], and dizziness [34]; however, all studies found no significant differences in the incidence of adverse events between the control and VR groups. Additionally, 2 studies reported no adverse events in 2 groups [27,28], whereas the other 6 studies did not report adverse events [23,24,29,31-33].

## Discussion

### Principal Findings

This study included 12 RCTs for meta-analysis and systematic review, with the aim of investigating the effectiveness and safety of VR technology during labor. We found that (1) the use of VR technology reduced maternal labor pain and anxiety, and that the effect on pain was influenced by whether exposure to VR was continuous or not; (2) there was no significant effect of the VR intervention on time to dilation of the uterine orifice in the first stage of labor or on the time to delivery of the fetus in the second stage of labor; and (3) VR used in maternal populations was safe.

#### VR Can Reduce the Labor Pain

The results of this review show that VR can relieve pain during childbirth. Considering the high heterogeneity among included studies, we performed a subgroup analysis, which demonstrated that the interruption of VR impacted pain reduction. It is possible that frequent interruptions, leading to less immersion in VR, diminished the distracting effect of VR for pain. We also surmised that increased maternal exposure to VR may reduce the novelty of this new technology, leading to a decrease in maternal interest in VR. This result highlights the importance of VR use at an appropriate frequency.

Regarding the active phase of labor, we performed a meta-analysis of 3 studies reporting that VR reduced pain levels, similar to the findings of other reviews of the application of VR for pain relief [27,30]. One study reported VR for epidural analgesia in labor and showed that pain relief was more pronounced in women using VR (*P*<.001) [25]. With the development of epidural techniques, the population of women undergoing such procedures in labor is likely to grow; therefore, further studies should be conducted in women undergoing epidural anesthesia to investigate its combined effect with VR and determine whether a synergistic effect can be achieved.

The VR interventions in the included studies used different contents and devices, and the intervention frequency also varied. One study found that different VR contents impacted the effect of the interventions; for example, natural landscapes could overlay with the effect of positive thinking interventions, whereas video contents, which combined visual and auditory stimuli in multiple ways, can increased the level of distraction; however, no evidence of which type of content was more effective was presented [29].

Notably, we did not identify any studies that used multidimensional tools to measure pain intensity during labor, and pain levels in the second and third stages of labor were unavailable, possibly because it was challenging to obtain accurate information under extreme conditions. Therefore, follow-up studies should focus on the timing and duration of VR use during delivery and explore the effect of different VR content on maternal delivery pain, to obtain the most effective intervention results.

#### VR Can Reduce the Labor Anxiety

We concluded that VR could relieve maternal anxiety during delivery, which is consistent with the findings of Eijlers et al [35], who found that applying VR could reduce anxiety in children; however, 1 study showed that the addition of VR for providing precesarean section information was not very effective in relieving anxiety [27], because anxiety is a persistent emotion influenced by various factors, such as lacking information, fear of pain, and worrying about the fetus [36]. Although VR can provide information visually over a short time and enhance understanding of the cesarean section, it cannot influence other sources of anxiety, such as concerns about pain and the fetus, and women undergoing planned cesarean section have a relatively longer time in which to obtain relevant information, regardless of whether they use VR. Therefore, it is essential to examine the most suitable time and appropriate populations in which to implement VR aimed at reducing maternal anxiety, to avoid the waste of resources.

#### VR Cannot Shorten the Labor Duration

Our review of the effect of VR on labor duration indicated that the difference in the effect of VR on the duration of the first and second stages of labor compared with the control group was not statistically significant. The labor process is influenced by a combination of psychological (anxiety, depression, fear, etc) [37] and physiological (pelvis, body mass, etc) factors [38]. VR technology only partially relieves pain and anxiety, since it involves distraction techniques [12], and thus has no statistically significant effect on labor duration. Nevertheless, some studies have shown that differences in the results were attributable to variation in the types of interventions and personal characteristics of women [33,34]; hence, further research on the effect of VR on labor duration is needed.

#### Satisfaction and Security of VR

According to our review, VR improved satisfaction at delivery, but most of the studies were not blinded to the subjects, which have led to bias; therefore, more RCTs blinded to subjects and investigators should be conducted in the future, to provide a basis for improving maternal satisfaction at delivery with VR.

The use of VR in maternal delivery is increasing, and the results of the review indicated that VR did not increase the incidence of adverse events during labor. In light of the small sample sizes and the lack of attention to long-term adverse effects, future studies should focus on the adverse effects of VR and lay the foundation for its standardized application in maternal delivery.

#### Strengths and Limitations

This is a comprehensive systematic review and meta-analysis of VR in maternal delivery. We used an exhaustive search strategy to facilitate full coverage of relevant studies and had detailed inclusion and exclusion criteria. The results of subgroup and sensitivity analyses suggested that the findings are robust. Furthermore, the studies included were conducted in ethnically diverse settings, expanding applicability.

However, this study has several limitations, due to demographic differences and clinical variations, as follows: (1) this systematic evaluation only searched studies published in Chinese and English, thus publication bias was presented due to the omission of gray studies; (2) variation in interventions and outcome indicators, as well as differences in maternal ethnicity and physical qualities among different countries, have influenced the outcomes; and (3) the low quality of the included studies and the limited sample size affected the accuracy of the results.

#### Explanation of Heterogeneity

Our meta-analysis detected a high degree of heterogeneity among trials. The heterogeneity across trials in the analysis of labor pain was considerable; therefore, we implemented subgroup and sensitivity analyses to further explore the sources of heterogeneity and found that, for pain during labor, the presence or absence of continuous VR intervention was an influencing factor. Second, the dispersion of the effects observed in the included trials could be due to unidentified fluctuations in labor pain, measurement method subjectivity, confusion, and the emotional stability of women in labor. For labor anxiety, differences in duration, schedule, intensity, and type of interventions and methodological factors have resulted in high heterogeneity. Differences among the observed effects on labor duration have also been due to the accuracy of determining the stage of labor and measurement methods. Additionally, other factors could have also contributed to the heterogeneity of labor pain, anxiety, and labor duration—for example, the experience of the mother in labor and the fetal condition.

#### Implications for Future Research and Practice

As a novel technology, VR provides considerable distraction effects, but it is still in the developmental stage, and the safety and cost-effectiveness of this approach for maternal use is debatable. It is recommended that future studies with larger sample sizes be conducted to verify the efficacy of VR. Second, based on the heterogeneity of intervention content and timing detected in this study, evidence is needed to provide fully substantiated recommendations regarding the frequency, duration, and content of VR interventions. Moreover, most of the reviewed study designs lacked theoretical support, and future studies should explore more formal models of VR intervention, to determine the optimal timing and effects of VR interventions and provide a more consistent and effective reference standard. Furthermore, the effects of VR interventions combined with epidural analgesia should be further explored, as epidural analgesia is becoming more common to reduce the abuse of analgesic drugs and their side effects. Finally, the studies did not report the long-term effects of VR use, and the measurement tools used were relatively subjective, which could have reduced their reliability. Future research could focus on (1) the construction of intervention models for VR use in labor; (2) the use of VR in combination with physiological indicators of labor, medications, etc; (3) more accurate intervention outcome measures; and (4) multicenter, large-sample, and high-quality study designs.

### Conclusion

Our review confirms that VR is effective and safe as a distraction intervention for relieving labor pain and anxiety; however, research on the use of VR in maternal labor is still in its infancy, and better designed and more rigorous large-scale RCTs are needed to provide a higher-quality evidence base for the use of VR technology in maternal labor, with the aim of improving labor experience and outcomes.

## Appendix

Multimedia Appendix 1 Search strategy.

## Abbreviations

MD: mean difference
PRISMA: Preferred Reporting Items for Systematic Reviews and Meta-Analyses
RCT: randomized controlled trial
SMD: standardized mean difference
VR: virtual reality
