# Supplementary material for: The Effects of Virtual Reality in Maternal Delivery: Systematic Review and Meta-analysis
Source: JMIR Serious Games. 2022 Nov 23;10(4):e36695. doi: 10.2196/36695 (PMC9730208; doi:10.2196/36695)
Supplement: Multimedia Appendix 1 [file games_v10i4e36695_app1.docx]

| Databases | Search Strategy |
| --- | --- |
| PubMed | (((((((((((("Pregnant Women"[Mesh]) OR (Woman, Pregnant[Title/Abstract])) OR (Women, Pregnant[Title/Abstract])) OR ("Delivery, Obstetric"[Mesh])) OR (Obstetric Deliveries[Title/Abstract])) OR (Obstetric Delivery[Title/Abstract])) OR ("Parturition"[Mesh])) OR (Parturitions[Title/Abstract])) OR (Birth[Title/Abstract])) OR (Births[Title/Abstract])) OR (Childbirth[Title/Abstract])) OR (Childbirths[Title/Abstract])) AND ((((((((((((((((((((((((((((((((((((((((("Virtual Reality"[Mesh] OR "Virtual Reality Exposure Therapy"[Mesh]) OR (Virtual Reality, Educational[Title/Abstract])) OR (Educational Virtual Realities[Title/Abstract])) OR (Educational Virtual Reality[Title/Abstract])) OR (Reality, Educational Virtual[Title/Abstract])) OR (Virtual Realities, Educational[Title/Abstract])) OR (Virtual Reality, Instructional[Title/Abstract])) OR (Instructional Virtual Realities[Title/Abstract])) OR (Instructional Virtual Reality[Title/Abstract])) OR (Realities, Instructional Virtual[Title/Abstract])) OR (Reality, Instructional Virtual[Title/Abstract])) OR (Virtual Realities, Instructional[Title/Abstract])) OR (Virtual Reality Immersion Therapy[Title/Abstract])) OR (Virtual Reality Therapy[Title/Abstract])) OR (Reality Therapies, Virtual[Title/Abstract])) OR (Reality Therapy, Virtual[Title/Abstract])) OR (Therapies, Virtual Reality[Title/Abstract])) OR (Therapy, Virtual Reality[Title/Abstract])) OR (Virtual Reality Therapies[Title/Abstract])) OR ("Augmented Reality"[Mesh])) OR (Augmented Realities[Title/Abstract])) OR (Realities, Augmented[Title/Abstract])) OR (Reality, Augmented[Title/Abstract])) OR (Mixed Reality[Title/Abstract])) OR (Mixed Realities[Title/Abstract])) OR (Realities, Mixed[Title/Abstract])) OR (Reality, Mixed[Title/Abstract])) OR ("User-Computer Interface"[Mesh])) OR (Virtual System[Title/Abstract])) OR (Interface, User-Computer[Title/Abstract])) OR (Interfaces, User-Computer[Title/Abstract])) OR (User-Computer Interfaces[Title/Abstract])) OR (Interfaces, User Computer[Title/Abstract])) OR (User Computer Interfaces[Title/Abstract])) OR (Interface, User Computer[Title/Abstract])) OR (Virtual Systems[Title/Abstract])) OR (System, Virtual[Title/Abstract])) OR (Systems, Virtual[Title/Abstract])) OR (virtual environment[Title/Abstract])) OR (immersion VR[Title/Abstract])) OR (Reality, Virtual[Title/Abstract]))  **Result:97(21.11.23)** |
| Embase | ('virtual reality exposure therapy'/exp OR 'computer interface'/exp OR 'augmented reality'/exp OR 'virtual reality'/exp OR 'augmented realities':ab,ti OR 'realities, augmented':ab,ti OR 'reality, augmented':ab,ti OR 'mixed reality':ab,ti OR 'mixed realities':ab,ti OR 'realities, mixed':ab,ti OR 'reality, mixed':ab,ti OR 'virtual system':ab,ti OR 'interface, user-computer':ab,ti OR 'interfaces, user-computer':ab,ti OR 'user-computer interfaces':ab,ti OR 'interfaces, user computer':ab,ti OR 'user computer interfaces':ab,ti OR 'interface, user computer':ab,ti OR 'virtual systems':ab,ti OR 'system, virtual':ab,ti OR 'systems, virtual':ab,ti OR 'virtual environment':ab,ti OR 'immersion vr':ab,ti OR 'reality, virtual':ab,ti OR 'virtual reality, educational':ab,ti OR 'educational virtual realities':ab,ti OR 'educational virtual reality':ab,ti OR 'reality, educational virtual':ab,ti OR 'virtual realities, educational':ab,ti OR 'virtual reality, instructional':ab,ti OR 'instructional virtual realities':ab,ti OR 'instructional virtual reality':ab,ti OR 'realities, instructional virtual':ab,ti OR 'reality, instructional virtual':ab,ti OR 'virtual realities, instructional':ab,ti OR 'virtual reality immersion therapy':ab,ti OR 'virtual reality therapy':ab,ti OR 'reality therapies, virtual':ab,ti OR 'reality therapy, virtual':ab,ti OR 'therapies, virtual reality':ab,ti OR 'therapy, virtual reality':ab,ti OR 'virtual reality therapies':ab,ti）AND （'pregnant woman'/exp OR 'obstetric delivery'/exp OR 'birth'/exp OR 'woman, pregnant':ab,ti OR 'women, pregnant':ab,ti OR 'deliveries, obstetric':ab,ti OR 'obstetric deliveries':ab,ti OR 'parturitions':ab,ti OR 'parturition':ab,ti OR 'births':ab,ti OR 'childbirths':ab,ti）  **Result:105(21.11.23)** |
| Web of Science | (((((((((((((((((((((TS=(" augmented reality" or "Augmented Realities" or "Realities, Augmented" or "Reality, Augmented" or "Mixed Reality" or "Mixed Realities" or "Realities, Mixed" or "Reality, Mixed")) OR TS=("User Computer Interface")) OR TS=("Virtual System")) OR TS=("Interface, User-Computer")) OR TS=("Interfaces, User-Computer")) OR TS=("User-Computer Interfaces")) OR TS=("Interfaces, User Computer")) OR TS=("User Computer Interfaces")) OR TS=("Interface, User Computer")) OR TS=("Virtual Systems")) OR TS=("System, Virtual")) OR TS=("Systems, Virtual")) OR TS=("virtual environment")) OR TS=("immersion VR")) OR TS=("Reality, Virtual")) OR TS=("Virtual Reality")) OR TS=("Virtual Reality, Educational")) OR TS=("Educational Virtual Realities" or "Educational Virtual Reality" or "Reality, Educational Virtual")) OR TS=("Virtual Realities, Educational" or "Virtual Reality, Instructional" or "Instructional Virtual Realities" or "Instructional Virtual Reality")) OR TS=("Realities, Instructional Virtual" or "Reality, Instructional Virtual" or "Virtual Realities, Instructional")) OR TS=("Virtual Reality Exposure Therapy" or "Virtual Reality Immersion Therapy" or "Virtual Reality Therapy" or "Reality Therapies, Virtual")) OR TS=("Reality Therapy, Virtual" or "Therapies, Virtual Reality" or "Therapy, Virtual Reality" or "Virtual Reality Therapies") And (TS=("Pregnant Woman" or "Woman, Pregnant" or "Women, Pregnant" or "Deliveries, Obstetric" or "Obstetric Deliveries" or "Obstetric Delivery")) OR TS=("Parturition" or "Parturitions" or "Birth" or "Births" or "Childbirth" or "Childbirths")  **Result:184(21.11.23)** |
| The Cochrane Library | ((MeSH descriptor: [Virtual Reality Exposure Therapy] explode all trees) or (MeSH descriptor: [Virtual Reality] explode all trees ) or (("virtual environment" or "immersion VR" or "Virtual Reality" or "Instructional Virtual Reality" or "Virtual Reality Immersion Therapy" or "Virtual Reality Therapy" or "Reality Therapy, Virtual" or "Therapy, Virtual Reality" or "augmented reality" or "Reality, Augmented" or "Mixed Reality" or "Reality, Mixed" or "User Computer Interface" or "Virtual System" or "Interface, User-Computer" or "User Computer Interfaces" or "System, Virtual"):ti,ab,kw )) and ((MeSH descriptor: [Pregnant Women] explode all trees) or (("Pregnant Woman" or "Woman, Pregnant" or "Women, Pregnant" or " Deliveries, Obstetric" or "Obstetric Delivery" or "Parturition" or "Birth" or "Childbirth"):ti,ab,kw ))  **Result:49(21.11.23)** |
| CINAHL | (SU (SU (pregnant woman or pregnant women or pregnancy or expecting mother ) OR SU women pregnancy OR SU Women, Pregnant OR SU Deliveries, Obstetric OR SU Obstetric Deliveries OR SU Obstetric Delivery OR SU Parturition OR SU Parturitions OR SU birth OR SU ( childbirth or labour or birth or labor or delivery or pregnancy ) OR SU Childbirths)) AND (SU ( (SU ( virtual reality or vr or Educational Virtual Reality ) OR SU reality virtuality OR SU ( virtual reality exposure therapy or or virtual reality therapy ) OR SU Virtual Reality Therapies OR SU Reality, Educational Virtual OR SU Virtual Realities, Educational OR SU Virtual Reality, Instructional OR SU Instructional Virtual Realities OR SU Instructional Virtual Reality OR SU Realities, Instructional Virtual OR SU Reality, Instructional Virtual OR SU Virtual Realities, Instructional) ) OR SU ( augmented reality or ar ) OR SU Reality, Augmented OR SU virtual systems OR SU mixed-reality simulation OR SU ( mixed reality or mr ) OR SU user-computer interface OR SU virtual environment OR SU immersion VR)  **Result:349(21.11.24)** |
| CKNI | ( (KY % '虚拟情景' OR KY % '虚拟情境' OR KY % '虚拟现实交互' OR KY % '人机交互' OR KY % ' 沉浸式体验' OR KY % '临境技术' OR KY % '混合现实 ' OR KY % '虚拟仿真技术' OR KY % '虚拟现实' OR KY % 'VR' OR KY % '增强现实') AND( KY % '孕妇' OR KY % '产妇' OR KY % '妊娠' OR KY % '分娩') ) OR ((SU %= '孕妇' OR SU %= '产妇' OR SU %= '妊娠' OR SU %= '分娩') AND (SU %= '虚拟现实' OR SU %= '虚拟情境' OR SU %= '虚拟情景' OR SU %= '虚拟现实交互' OR SU %= '人机交互' OR SU %= '沉浸式体验' OR SU %= '临境技术' OR SU %= '混合现实' OR SU %= '虚拟仿真' OR SU %= 'VR' OR SU %= '增强现实') )同义词扩展+网络首发+增强出版  **Result:23(21.11.24)** |
| Wan-Fang database | (主题:(虚拟情景) or 主题:(虚拟情境) or 主题:(虚拟现实交互) or 主题:(人机交互) or 主题:(沉浸式体验) or 主题:(临境技术) or 主题:(混合现实) or 主题:(虚拟仿真) or 主题:(虚拟现实) or 主题:(VR) or 主题:(增强现实) ) and (主题:(孕妇) or 主题:(产妇) or 主题:(妊娠) or 主题:(分娩) )  **Result:337(21.11.24)** |
